# Phenotyping for drought tolerance of crops in the genomics era

**DOI:** 10.3389/fphys.2012.00347

**Published:** 2012-09-19

**Authors:** Roberto Tuberosa

**Affiliations:** Department of Agroenvironmental Science and Technology, University of BolognaBologna, Italy

**Keywords:** drought tolerance, phenomics, genomics, QTL, breeding, yield, phenology, modeling

## Abstract

Improving crops yield under water-limited conditions is the most daunting challenge faced by breeders. To this end, accurate, relevant phenotyping plays an increasingly pivotal role for the selection of drought-resilient genotypes and, more in general, for a meaningful dissection of the quantitative genetic landscape that underscores the adaptive response of crops to drought. A major and universally recognized obstacle to a more effective translation of the results produced by drought-related studies into improved cultivars is the difficulty in properly phenotyping in a high-throughput fashion in order to identify the quantitative trait loci that govern yield and related traits across different water regimes. This review provides basic principles and a broad set of references useful for the management of phenotyping practices for the study and genetic dissection of drought tolerance and, ultimately, for the release of drought-tolerant cultivars.

## Introduction

Crops are exposed to the ravages of drought in various ways and to different extents. Regrettably, global climate change will increase the occurrence and severity of drought episodes, not least due to the higher evapotranspirative demand created by rising temperatures. Altogether, these changes have already been shown to offset a significant portion of the increases in average yields that during the past three decades arose from technology, CO_2_ fertilization and other factors (Lobell et al., 2011). Therefore, food security in the twenty-first century will rely increasingly on the release of cultivars with improved resistance to drought conditions and with high yield stability (Swaminathan, 2005; Borlaug, 2007; Pennisi, 2008; Luo, 2010; Tester and Langridge, 2010; Reynolds et al., 2011; Serraj et al., 2011; Chapman et al., 2012).

In this challenging scenario, molecular approaches offer novel opportunities for the dissection and more targeted manipulation of the genetic and functional basis of yield under drought conditions (Forster et al., 2000; Sinclair et al., 2004; Bohnert et al., 2006; Mackill, 2006; Tuberosa and Salvi, 2006; Jenks et al., 2007; Nelson et al., 2007; Ortiz et al., 2007a; Vij and Tyagi, 2007; Leung, 2008; Xu and Crouch, 2008; Ashraf, 2010; Mittler and Blumwald, 2010; Yadav et al., 2011; Deikman et al., 2012). Additionally, the “-omics” platforms now allow for extensive mining of the transcriptome (Rabbani et al., 2003; Poroyko et al., 2007; Degenkolbe et al., 2009; Ergen and Budak, 2009; Sreenivasulu et al., 2010; Deokar et al., 2011; Hiremath et al., 2011), metabolome (Fernie and Schauer, 2009) and proteome (Timperio et al., 2008). Although, some may not consider “-omics” data as phenotypes *sensu stricto*, they should be treated as such, considering that they represent crucial steps that are progressively removed from genes to their ultimate phenes (Houle et al., 2010; Furbank and Tester, 2011). Not with standing the deluge of molecular data produced in the past decade, the applicable results reported so far with non-conventional approaches have not met expectations (Edmeades et al., 2004; Araus et al., 2007, 2008; Collins et al., 2008; Xu and Crouch, 2008; Heffner et al., 2009; Passioura, 2010; Sinclair, 2011), partly because the progress in high-throughput, quality phenotyping has lagged behind.

Before analyzing the factors that affect the quality of phenotypic data collected under water-limited conditions, it is important to define the nomenclature and mechanisms of crop adaptation to drought and clarify their functional basis. Most of the examples and references provided in this review refer to cereals, which, as compared to other crops, have been more extensively investigated under drought conditions. Nevertheless, most concepts presented herein are equally valid for other crops as well.

## Drought adaptation: concepts, nomenclature, and mechanisms

In agriculture, the term “drought” refers to a condition in which the amount of water available through rainfall and/or irrigation is insufficient to meet the transpiration needs of the crop. The examples presented in this review provide some general guidelines on the different mechanisms that allow plants to withstand and eventually mitigate the negative effects of water deficit. In general, a clear distinction should be made between traits that help plants to survive a severe drought stress and traits that mitigate yield losses in crops exposed to a mild or intermediate level of water stress. Modern breeding activities, including phenotyping conditions, have predominantly targeted the latter levels of stress. Although, yield remains an elusive and neglected concept in most molecular studies carried out under water-limited conditions, it is an appropriate way to gauge the overall phenotypic value of any accession.

### The functional basis of drought resistance

Among the several definitions of drought resistance that have been provided during the past decades, the original one formulated by Levitt (1972) retains its validity and offers a rational approach to classify the strategies that allow plants to mitigate the negative effects of water deficit. Levitt (1972) classified the different mechanisms or strategies of drought resistance into two broad categories: dehydration avoidance and dehydration tolerance. In this respect, morpho-physiological features [e.g., deep roots, early flowering, deposition of epicuticular waxes, osmotic adjustment (OA), etc.] that enable the plant, or parts thereof, to maintain hydration are classified under dehydration avoidance. Conversely, features (e.g., remobilization of stem water-soluble carbohydrates (WSC), accumulation of molecular protectants, etc.) that allow the plant to maintain, at least partially, proper functionality in a severely dehydrated state are classified under dehydration (desiccation) tolerance. Carefully planned experiments conducted under controlled conditions allow us to separate the action of loci imparting avoidance from those providing tolerance to drought (Yue et al., 2006). Several reviews and dedicated volumes have addressed the mechanisms underlying drought resistance and the strategies that can improve yield under such conditions (Blum, 1988, 1996, 2009, 2011; Ludlow and Muchow, 1990; Ceccarelli and Grando, 1996; Passioura, 1996, 2007, 2010; Richards, 1996; Turner, 1997; Ribaut, 2006; Fischer et al., 2003; Boyer and Westgate, 2004; Chaves and Oliveira, 2004; Tuberosa, 2004; Araus et al., 2008; Kumar et al., 2008; Morison et al., 2008; Reynolds and Tuberosa, 2008; Farooq et al., 2009; Passioura and Angus, 2010; Yang et al., 2010; Sadok and Sinclair, 2011; Sinclair, 2011; Cairns et al., 2012; Mir et al., 2012).

The first step is to define the population of environments to be targeted, also identified as the TPE (target population of environments). Differences in TPE are largely determined by long-term patterns of genotype-by-environment interactions (GEI). The identification and characterization of a TPE is facilitated by the use of crop simulation models based on historic records of weather data. Simulation can describe a TPE by the frequency of occurrence of specific abiotic stresses and be based on the soil moisture profile along the crop cycle (Chapman et al., 2003). In Mediterranean environments, wheat and barley usually experience terminal drought caused by high temperatures during the grain-filling period (Araus et al., 2008). Nevertheless, within each TPE and GEI are frequently observed relating to yearly fluctuations in environmental factors (e.g., rainfall, temperature, etc.), diseases (e.g., foliar disease), and/or parasites (e.g., insects). Ideally, phenotyping for drought tolerance and yield stability should be carried out across a broad range of environments present within the TPE. During past decades, these multienvironment trials have been instrumental in increasing yield potential and also in maintaining yield stability under drought-stressed conditions in temperate maize (Tollenaar and Wu, 1999; Duvick, 2005; Tollenaar and Lee, 2006) as well as in other crops (Lafitte et al., 2006; Crossa et al., 2007; Acuna et al., 2008). In a few cases, they have also allowed for the identification of major QTLs consistently affecting yield across a range of water availability (Bernier et al., 2007, 2009; Maccaferri et al., 2008; Venuprasad et al., 2009a,b, 2012; Vikram et al., 2011; Dixit et al., 2012; Ghimire et al., 2012).

### Water-use efficiency and grain yield under water-limited conditions

Water-use efficiency (WUE) is the amount of dry matter produced [grain yield (GY) in the case of grain crops when considering seasonal WUE] per unit of water lost through evapotranspiration. A classical formula that highlights the critical role of WUE in determining GY in crops grown in water-limited conditions was suggested by Passioura (1977):

GY=W×WUE×HI

where W is the total amount of water transpired by the crop and evaporated from the field and HI is the harvest index, i.e., the ratio between GY and total biomass. Salekdeh et al. (2009) identify phenotyping protocols that address each formula's factors, describe their key features and illustrate their integration with different molecular approaches. When using this formula, one should consider the possible interdependence of these variables, with the result that selection for improving WUE in order to increase GY may be partially counterbalanced by a reduction in the amount of water extracted from the soil. In fact, a number of traits influence both W and WUE.

The most important factor is matching the phenological development pattern of the crop and the seasonal rainfall pattern (Richards, 1996; Turner, 1997; Araus et al., 2003; Morison et al., 2008; Soltani and Sinclair, 2012). Early vigor potentially improves both W and WUE, while deep roots and/or osmoregulation under appropriate conditions increase water extraction from the soil (Blum, 1988, 2011; Ludlow and Muchow, 1990; Richards, 2006; Reynolds and Tuberosa, 2008; Sadok and Sinclair, 2011). However, we should keep in mind that farmers eventually harvest grain and not WUE, which means that a lower WUE may actually be desirable when WUE is negatively associated with GY, as is well-known in cereals differing in their intrinsic WUE (Blum, 2005, 2006, 2009). Therefore, WUE should not be equated to drought tolerance. The best example is provided by a population of related progeny such as recombinant inbred lines (RILs) that differ in their capacity to extract soil moisture due to differences in root depth, and hence greater capacity to access moisture stored in deeper soil layers. Because, WUE is higher in genotypes characterized by low stomatal conductance, often resulting from a lower water status, the genotypes that are more wasteful (i.e., with a lower WUE) and able to extract more water from the soil (Merah, 2001; Rebetzke et al., 2002; Blum, 2006, 2009, 2011), whilst maintaining higher stomatal conductance, will have higher yield. Conversely, under conditions of limited soil moisture, low WUE resulting from excessive evapotranspiration will not allow sustained accumulation of dry matter and its partitioning to reproductive organs (Monneveux and Ribaut, 2006; Richards, 2006; Tambussi et al., 2007; Barnabas et al., 2008; Sinclair et al., 2008). This finding introduces an essential concept for interpreting cause–effect relationships between morphophysiological traits and GY under drought conditions: the sign and magnitude of this relationship at the whole-plant or QTL level are not universal and can change widely according to the dynamics (i.e., frequency and timing) and intensity of the drought episode/s (Collins et al., 2008; Sinclair et al., 2010; Tardieu, 2012).

An alternative formula to address properly the factors influencing WUE in crops grown under water-limited conditions has been proposed by Richards (1991):

$$\text{WUE (biomass)}=\text{TE}/(1+E_{s}/T)$$

where TE is the transpiration efficiency (above ground dry weight/transpired water), *E*_*s*_ is the water lost by evaporation from the soil surface and *T* is water lost through transpiration by the crop. Analysis of the variables in this formula provides a useful framework for identifying the agronomic and breeding strategies, and hence phenotyping targets, most suitable for optimizing WUE and maximizing yield in environments that differ in rainfall distribution during the crop cycle.

At the leaf level, “intrinsic WUE” indicates the ratio of the instantaneous rates of CO_2_ assimilation and stomatal transpiration. Condon et al. (2002) discussed the factors influencing intrinsic WUE and how an increased intrinsic WUE can be achieved through either lower stomatal conductance, higher photosynthetic capacity, or both. The same authors caution about the possible penalties in terms of yield through manipulation of each variable. They conclude that to achieve more widespread gains in cereal yield derived from greater intrinsic WUE, it is necessary to decouple intrinsic WUE and low crop growth rate. In practical terms, WUE becomes more important when crops grow predominantly on stored soil moisture (Condon et al., 2002), as reflected by the release of wheat cultivars Drysdale and Rees (Richards, 2006), specifically selected for target areas where wheat is grown under such conditions.

## Which traits should be targeted?

The morphophysiological traits and the corresponding QTLs that affect yield in drought conditions can be categorized as constitutive (i.e., also expressed under well-watered conditions) or drought-responsive (i.e., expressed only under pronounced water shortage; Lafitte and Edmeades, 1995; Blum, 2006). While drought-responsive traits/QTLs usually affect yield only under rather severe drought conditions, constitutive traits/QTLs can affect yield at low and intermediate levels of drought stress as well. The response of QTLs for drought-adaptive traits (e.g., accumulation of osmolytes, relocation of WSC, etc.) to drought is probably due to regulation of the expression of the underlying structural genes in response to signaling cues such as abscisic acid (ABA) accumulation (Bray, 2002) that are reinforced by cellular dehydration. Under appropriate soil moisture conditions, the presence of QTLs for traits usually classified as constitutive but difficult to measure (e.g., root depth) can be revealed by the collocation of QTLs for traits indicative of the water status of the plant such as ABA concentration, stomatal conductance, canopy temperature depression (CTD), etc., (Lebreton et al., 1995; Tuberosa et al., 2002b; Reynolds et al., 2009, 2011). Experimental evidence indicates that the progress achieved by breeders during the last century can mainly be accounted for by changes in constitutive traits that affect dehydration avoidance rather than drought-responsive traits (Blum, 2005, 2006, 2011). In this respect, emphasis is increasingly being placed on phenotyping traits that constitutively enhance yield *per se* (Blum, 2009; Passioura, 2010), rather than on characteristics that enhance plant survival under extreme drought (Bartels et al., 2006), in view of a possible negative trade-off under less severe circumstances (Blum, 1996, 2005, 2006; Passioura, 2002, 2007, 2010; Sinclair, 2011).

The traits to be considered as potential selection targets for improving yield under water-limited conditions must be genetically (i.e., causally) correlated with yield, and should have a greater heritability than yield itself (Blum, 1988, 2011; Monneveux and Ribaut, 2006). Additional desirable features are the presence of sufficient genetic variability and lack of yield penalties under favorable conditions. Ideally, measurement of the target trait should be non-destructive, rapid, accurate, and inexpensive. It should also be possible to measure the trait using a small number of plants and without lengthy procedures to calibrate sensors to individual plants. Finally, rather than reporting on short-term features at the cellular level, the nature of the secondary trait should be integrative across the growing cycle, or part thereof, and relate to higher levels of functional organization (e.g., the canopy level rather than the single leaf), thereby providing information on the long-term ecophysiological performance of the crop. General information and examples are now provided on a number of traits that have been investigated for their influence on drought resistance and/or WUE.

### Early vigor

Early vigor under conditions of low evapotranspiration may allow annual crops to optimize WUE and limit the loss of water due to direct evaporation from the soil surface. This leaves more stored water available for later developmental stages when soil moisture becomes progressively exhausted and increasingly limiting for yield (Slafer et al., 2005; Richards, 2006; Rebetzke et al., 2007; Richards et al., 2007). Early establishment also reduces the occurrence of inhibition of stomatal conductance as a consequence of root-borne signaling such as from ABA through the xylem flow (Davies et al., 2000; Ren et al., 2007) caused by shallow and superficial roots (Blum, 1996; Giuliani et al., 2005). As a trade-off, excessively vigorous canopy development may cause early depletion of soil moisture. The optimal degree of vigor will thus depend on the environmental characteristics of the TPE. Early vigor has been exploited to improve WUE and yield in wheat (Asseng et al., 2003; Richards, 2006; Rebetzke et al., 2007). QTLs for the growth rate of wheat seedlings (Spielmeyer et al., 2007) are being targeted at CSIRO (Commonwealth Scientific and Industrial Research Organization, Australia)^1^.

### Root architecture

Roots exhibit an astounding level of morphological plasticity in response to soil physical conditions (Passioura, 1983; Bengough et al., 2006; Gerald et al., 2006; Ito et al., 2006; Kato et al., 2007; Lynch, 2007; Forde, 2009; Siopongco et al., 2009), a peculiarity that allows plants to adapt better to the chemical and physical properties of the soil, particularly under drought conditions (Bacon et al., 2002; Yu et al., 2007). The concept of root ideotype should be elaborated only after gaining a detailed understanding of: (1) the factors that limit the availability of soil moisture to the crop; and (2) the metabolic cost sustained by the plant to develop and maintain a more vigorous root system. Notably, recurrent selection for increased GY in drought-stressed tropical maize was associated with a decrease in root mass (Bolaños and Edmeades, 1993). Accordingly, the effects of root size and architecture on final yield will depend on the distribution of soil moisture and the level of competition for water resources within the plant community (King et al., 2009). Therefore, when additional stored moisture is available in deeper soil layers, selection for faster growing and deeper roots could enhance water harvest and help stabilize yield under drought conditions.

The importance of a deep and vigorous root system for higher yield has been recognized in bean (Mohamed et al., 2002), soybean (Sadok and Sinclair, 2011), chickpea (Varshney et al., 2011), lettuce (Johnson et al., 2000), maize (Tuberosa et al., 2003, 2007b, 2011b; Hammer et al., 2009; Landi et al., 2010; Hund et al., 2011), barley (Forster et al., 2005), wheat (Manschadi et al., 2006, 2010; Wasson et al., 2012), and especially, in rainfed rice (Nguyen et al., 1997; Price and Tomos, 1997; Ali et al., 2000; Babu et al., 2003; Courtois et al., 2003, 2009; Steele et al., 2006, 2007; Kamoshita et al., 2008; Witcombe et al., 2008; Bernier et al., 2009; Henry et al., 2011). However, other experiments in rice have shown a lack of correlation between root features and drought resistance (Pantuwan et al., 2002; Subashri et al., 2009).

The main drawback to the study of root features and their use as selection criteria relates to the difficulty of phenotyping field-grown plants (Richards, 2008). A number of techniques allow for the estimation of root mass and its distribution in the soil profile. These techniques require different amounts of labor and plot destruction for sample collection. The fastest but most destructive technique measures the vertical pulling strength required to uproot the plant, as a proxy for root mass and architecture (Lebreton et al., 1995; Sanguineti et al., 1998; Landi et al., 2002). Recently, a high-throughput, albeit equally destructive approach also known as “shovelomics,” has been deployed to investigate several root architectural features in field-grown maize (Trachsel et al., 2011). Other less destructive but much more time-consuming techniques such as excavation and coring methods have also been used to estimate root mass and distribution (Nissen et al., 2008).

Minirhizotrons provide a non-destructive, *in situ* method for directly viewing and studying fine roots (Johnson et al., 2001; Smit and Groenwold, 2005). Tube installation is critical, and steps must be taken to ensure good soil/tube contact without compacting the soil. Tube installation causes some degree of soil disturbance and has the potential to create artifacts in root data collection and analysis, resulting in biased values. Therefore, a waiting period of a few months between tube installation and image collection is recommended to allow roots to recolonize the space around the tubes and to permit nutrients to return to pre-disturbance levels (Johnson et al., 2001). The frequency of image collection depends upon the root parameters being measured or calculated, and the time and resources available for collecting images and extracting data.

In maize, a fast non-destructive method to estimate root mass has relied on the use of a hand-held capacitance meter (van Beem et al., 1998; McBride et al., 2008). The accuracy of this method was tested by comparing the results with direct measurements taken on uprooted plants grown in the greenhouse and in the field. The significant correlation (*r* from 0.56 to 0.73) between the methods suggests the feasibility of using capacitance meters for routine, non-destructive observations repeated over time. Despite this possibility, the method has not been widely applied.

Heterogeneity in soil structure and composition hinders the acquisition of accurate values for root features in field-grown plants. As an alternative to root phenotyping in field experiments, a number of studies have measured roots in plants grown under controlled conditions (Arihara and Crosbie, 1982; Price et al., 1997a,2002b,c; Landi et al., 1998, 2001a; Tuberosa et al., 2002b; de Dorlodot et al., 2005, 2007; Kimurto et al., 2005; Zhu et al., 2006, 2011; Hochholdinger and Tuberosa, 2009; Zaman-Allah et al., 2011a; Ren et al., 2012). This allows more rapid and accurate analysis of root features. A major shortcoming of these studies is the unnatural environment in which the roots grow, suggesting great caution in extrapolating the results to field-grown plants. In maize, a significant, albeit weak, positive association has been reported between seminal root traits in hydroponics and root pulling resistance in the field (Landi et al., 2001a; Tuberosa et al., 2002b). A reasonable compromise to avoid both the unnatural conditions present in hydroponics and/or aeroponics and the difficulty of studying roots in the field is offered by growing plants in pots, columns and/or observation chambers filled with soil (Azhiri-Sigari et al., 2000; Wade et al., 2000; Zaman-Allah et al., 2011a). Pot experiments also allow for a precise measurement of the amount of water provided to each plant, hence water use and WUE (Price et al., 2002b), and to estimate the capacity of roots to penetrate a wax layer of high mechanical impedance mimicking a soil hardpan, often the main constraint that limits access of roots to soil moisture in deeper soil layers (Cairns et al., 2004; Nhan et al., 2006; Acuna et al., 2007). In rice, an enhanced capacity to penetrate a soil hardpan is considered an essential feature for the development of deeper roots under rainfed lowland conditions (Fukai and Cooper, 1995) and is a key factor in drought adaptation in areas where water supply is limited (Siopongco et al., 2009).

Gel- or soil-filled chambers, soil sacs, pouches, paper rolls, X-ray microtomography, and magnetic resonance imaging (MRI) have also been used to investigate bi- and tri-dimensional root architecture (Bengough et al., 2004; Sanguineti et al., 2007; Hargreaves et al., 2009; Norton and Price, 2009; Ruta et al., 2010; Tracy et al., 2010; Bovina et al., 2011; Clark et al., 2011; Rascher et al., 2011; Singh et al., 2011; Alhosein et al., 2012; De Smet et al., 2012; Hamada et al., 2012; Mace et al., 2012). These experiments are particularly suited to the discovery of QTLs that are prevalently expressed in a constitutive fashion and which, as such, are more likely to influence root architectural features (e.g., root angle) across different soil conditions.

### Flowering time

Flowering time is recognized as the most critical factor to optimize adaptation, hence yield, in environments differing in water availability and distribution during the growing season (Richards, 2006). Positive associations between plasticity of yield and flowering time across different levels of water availability have been reported in different crops (Sadras et al., 2009). Therefore, in addition to phenology *per se* (i.e., mean time to a phenological stage), plasticity of phenological development merits consideration as a distinct trait influencing crop adaptation and the outcome of any QTL experiment where the effects of phenology on yield are not duly recognized and accounted for (Pinto et al., 2010; Sabadin et al., 2012).

Many studies have investigated the genetic basis of flowering time, reflecting the economic importance of this trait. In annual crops, the genetic basis of flowering time is more complex in temperate species (e.g., barley, wheat, rye, etc.) as compared to tropical species (e.g., rice, sorghum, maize, etc.), due to the presence in the former group of verbalization genes influencing flowering time in response to low temperatures. In cereals, the switch from the vegetative to the reproductive phase is controlled, according to the species, by several genes responsive to verbalization and/or daylength as well as by loci for earliness *per se* (Salvi et al., 2002, 2007, 2011; Distelfeld et al., 2009).

In maize, a valuable selection target for improving drought resistance is provided by the anthesis-silking interval (ASI), a trait of intermediate heritability that is usually negatively correlated with GY under drought conditions (Bolaños and Edmeades, 1996; Monneveux and Ribaut, 2006). Because ASI can be phenotyped quite easily and effectively under the right experimental conditions, substantial breeding efforts have targeted this trait through conventional breeding (Chapman and Edmeades, 1999) or, once QTLs have been identified (Ribaut et al., 1996; Li et al., 2003a; Hao et al., 2008), with marker-assisted selection (MAS) (Ribaut et al., 2004; Ribaut and Ragot, 2007). The negative association reported between the effects of QTLs that have been shown to influence both leaf elongation and ASI suggests turgor maintenance as a possible common mechanism accounting for the correlation (Welcker et al., 2007).

### Carbon isotope discrimination

Carbon isotope discrimination (Δ^13^C) measures the ratio of stable carbon isotopes (^13^C/^12^C) in the plant dry matter compared to the ratio in the atmosphere (Condon et al., 1990). Because of differences in leaf anatomy and the mechanisms of carbon fixation in species with the C_3_ or C_4_ pathway, studies on Δ^13^C have wider implications for C_3_ species where the variation in Δ^13^C is larger than in C_4_ species and has a greater impact on crop yield (Condon et al., 1990, 2006). Commonly, but not always (Turner et al., 2007), Δ^13^C is negatively associated with WUE over the period of dry mass accumulation (Condon et al., 1990, 2004; Araus et al., 2002; Rebetzke et al., 2002; Xu et al., 2007; Royo et al., 2008).

Under drought stress, Δ^13^C is a good predictor of stomatal conductance (Condon et al., 2002) and WUE in different crops (Turner, 1997; Tambussi et al., 2007). A number of studies conducted in bread wheat under varying conditions of water availability have shown that the correlation between Δ^13^C and final GY varies from positive, when ample water is available to the crop, to negative in drought conditions, with no correlation at all in intermediate conditions (Condon et al., 1993, 2004). These results can be interpreted based on the influence of both stomatal conductance and photosynthetic activity on Δ^13^C, and on the fact that biomass production is limited in wet years by a lower stomatal conductance—an advantage under drought conditions (Turner, 1997). Δ^13^C measured in grains correlates positively with growth cycle duration (Araus et al., 1997) and negatively with leaf temperature (Richards et al., 2002). Therefore, the relationship between Δ^13^C and GY depends on the environmental conditions, the phenology of the crop and the plant organ (e.g., leaf or grain) from which the samples are collected (Araus et al., 1997; Merah et al., 2001; Condon et al., 2004).

High genetic variation for grain Δ^13^C has been reported in C_3_ species (Turner, 1997; Chen et al., 2012), with high heritability (e.g., from 0.76 to 0.85 in durum wheat; Merah et al., 2001) and a low GEI (Richards, 1996; Rebetzke et al., 2008a). For these characteristics, Δ^13^C is an attractive breeding target for improving WUE and yield, while the high cost required to measure each sample makes it an interesting candidate for MAS.

### Stomatal conductance

Stomatal conductance plays a pivotal role in regulating the water balance of the plant and determining Δ^13^C and WUE (Condon et al., 2002; Richards et al., 2002, 2007; Sinclair et al., 2008, 2010). A retrospective study conducted by Fischer et al. (1998) on a historical series of successful bread wheat cultivars released by CIMMYT from 1962 to 1988 showed a strong positive correlation between stomatal conductance and GY (*r* = 0.94; Fischer et al., 1998), indicating the possibility of raising the yield potential, hence the amount of water used by the crop, through an indirect selection for stomatal conductance and/or leaf temperature.

Given the laborious nature of measuring stomatal conductance, identifying the corresponding QTLs would allow for the implementation of MAS. In fact, it is difficult to accurately measure stomatal conductance in a reasonably large number of plants while properly accounting for the fluctuation in the main environmental factors known to affect stomatal conductance during the day (wind, solar radiation, humidity, etc.). A number of studies have reported QTLs for stomatal conductance (Lebreton et al., 1995; Price et al., 1997b, 2002a; Sanguineti et al., 1999; Ulloa et al., 2000; Takai et al., 2006; Khowaja and Price, 2008).

A more attractive and integrative way to indirectly monitor stomatal conductance through an extended time-period is based on the measurement of the natural oxygen isotope composition (d^18^O) in leaf and grain materials (Barbour et al., 2000; Ferrio et al., 2007). Compared with stomatal conductance, measuring d^18^O in plant material offers four advantages: (1) it provides an integrated measure of stomatal conductance and leaf temperature over the period that the analyzed tissue was formed; (2) it avoids a number of experimental problems typical of measuring stomatal conductance; (3) it allows for the collection of a large number of samples, and (4) requires very little labor in the field. In the historical series of CIMMYT wheat cultivars tested under irrigated conditions (see above), leaf d^18^O was strongly correlated with stomatal conductance (*r* = −0.93; Barbour et al., 2000). In this case, GY was more strongly correlated with leaf d^18^O (*r* = −0.90) as compared to leaf d^13^C (*r* = −0.71). However, the authors caution that d^18^O is a questionable yield predictor when stomatal conductance and GY are not strongly correlated.

### Canopy temperature depression

CTD as measured by thermal imaging is the difference in temperature between the canopy surface and the surrounding air. CTD is a highly integrating trait resulting from the effects of several biochemical and morphophysiological features acting at the root, stomata, leaf, and canopy levels. In the field, genotypes with a cooler canopy temperature under drought stress, or a higher CTD, use more of the available water in the soil to avoid excessive dehydration (Blum, 1988; Ludlow and Muchow, 1990; Reynolds et al., 2007, 2009). Infrared thermometry can report subtle differences in leaf temperature in both field and controlled conditions (Blum et al., 1982; Jones et al., 2003, 2009; Chaerle et al., 2007; Winterhalter et al., 2011a,b). Importantly, data collection is fast and non-destructive.

CTD is useful mainly in hot and dry environments typical of countries with a Mediterranean climate. Measurements should preferably be made on recently irrigated crops on cloudless and windless days with high vapor pressure deficits. Under these conditions and provided that data are collected when the canopy is sufficiently expanded to cover the soil, CTD can be a good predictor of wheat GY (*r* = 0.6–0.8; Reynolds and Pfeiffer, 2000). In bread wheat, yield progress was found to be associated with cooler canopies (Fischer et al., 1998) and significant genetic gains in yield have been reported in response to direct selection for CTD (Reynolds et al., 1999, 2009; Brennan et al., 2007). The addition of CTD as a selection criterion in wheat nursery improved considerably the identification of the highest yielding materials (van Ginkel and Ogbonnaya, 2007). These results are in keeping with the conclusions of Olivares-Villegas et al. (2007): “*Canopy temperature epitomises a mechanism of dehydration avoidance expressed throughout the cycle and across latitudes, which can be utilized as a selection criterion to identify high-yielding wheat genotypes or as an important predictor of yield performance under drought*.”

Grant et al. (2006) investigated the robustness and sensitivity of thermal imaging for detecting changes in stomatal conductance and leaf water status in a range of plant species (grapevine, bean and lupin) under greenhouse or controlled environment conditions. In particular, they compared absolute leaf temperatures and thermal indices of plant stress with stomatal conductance and water potential. Thermal imaging successfully distinguished between irrigated and non-irrigated plants of different species, with strong correlations between thermal indices and stomatal conductance as measured with a leaf pyrometer. Their results also highlighted factors such as leaf angle that should be addressed when using thermal imaging for indirect measurement of the level of drought stress of the tested materials. Additionally, these results are valuable for the design of protocols for application in crop production or ecosystem monitoring.

### Abscisic acid concentration

One of the main factors influencing leaf temperature via an effect on transpiration through stomatal conductance is the concentration of ABA in the leaf tissue and, ultimately, in guard cells (Wasilewska et al., 2008; Sirichandra et al., 2009). Therefore, ABA is a fundamental component of the mechanisms allowing the plant to match the water demand with the water supply and to optimize growth and survival in response to both daily and more long-term environmental fluctuations (Zhang and Davies, 1990; Xiong et al., 2007). Indeed, an increase in ABA concentration is a universal response observed in plants subjected to drought and other abiotic stresses (Quarrie, 1991; Setter, 2006). Additionally, ABA modulates the expression of a large number of genes whose products protect the cell from the harmful effects of dehydration (Bray, 2002; Seki et al., 2007).

ABA has been shown to affect many of the traits that influence the water balance of the plant through both dehydration avoidance and dehydration tolerance (Thompson et al., 2007). In maize seedlings subjected to artificially induced conditions of water deprivation, an increased ABA concentration enhanced the root/shoot ratio (Spollen et al., 2000; Sharp, 2002; Sharp et al., 2004), an adaptive change beneficial for increasing water uptake. It has also been shown that ABA facilitates water uptake into roots as the soil begins to dry, particularly under non-transpiring conditions, when the apoplastic path of water transport is largely excluded (Hose et al., 2001). Under terminal drought, tolerant pearl millet [*Pennisetum glaucum* (L.) R. Br.] have high leaf ABA and reduced transpiration at high vapor pressure deficit, a feature that highlights the important role of constitutive water-conserving mechanisms in maximizing yield under such conditions (Kholova et al., 2010a,b). The positive role on yield of a conservative water use, rather than deep or profuse rooting, has also been highlighted in chickpea (Zaman-Allah et al., 2011a,b).

In cereals, an accumulation of ABA has been implicated as one of the factors that influence reproductive fertility (Saini and Westgate, 2000; Landi et al., 2001b; Setter et al., 2001; Boyer and Westgate, 2004; McLaughlin and Boyer, 2007; Yang et al., 2007; Tang et al., 2008; Zhang et al., 2009) and endosperm development (Ober et al., 1991; Tuberosa et al., 1992; Setter et al., 1996; Mambelli and Setter, 1998; Seiler et al., 2011). In rice, selection for reduced ABA root signaling has been advocated as a means for better exploitation of subsoil water under mild or transient water deficit (Siopongco et al., 2008, 2009).

Sensitivity to ABA is also of interest for its implications on the adaptive response of plants to drought (Cominelli et al., 2005). Genetic variability for sensitivity to ABA has been reported in maize (Frascaroli and Tuberosa, 1993). Gametophytic selection carried out by spraying maize silks with an ABA solution before pollination led to significant effects on early vigor and other agronomic traits (Frascaroli and Landi, 1996; Landi et al., 2000).

Due to the availability of ABA-specific monoclonal antibodies (Quarrie et al., 1988) that allow for the cost-effective measurement of a large number of samples, several studies have been devoted to the identification of QTLs for ABA concentration and the analysis of their associated effects on other drought-related traits and yield (Lebreton et al., 1995; Tuberosa et al., 1998, 2002a; Sanguineti et al., 1999; Reymond et al., 2003; Giuliani et al., 2005; Landi et al., 2005, 2007; Rahman et al., 2011). Altogether, these studies do not provide a unifying picture of the role of ABA in determining yield, perhaps not unexpectedly in view of the different species and genetic backgrounds involved. Nevertheless, it is worth noting that the evaluation of an historical series of maize hybrids released in the past 60 years has shown a significant decrease in the capacity to accumulate ABA in response to a given level of water stress (Sanguineti et al., 2006) and, consequently, a negative correlation (*r* = −0.62) between the capacity to accumulate ABA at the seedling stage (a trait never selected for by breeders) and GY.

### Osmotic adjustment

OA is a metabolic process entailing a net increase in intercellular solutes in response to water stress (Morgan, 1984; Zhang et al., 1999; Serraj and Sinclair, 2002). As soil moisture declines, OA favors turgor maintenance, and hence the integrity of metabolic functions. Importantly, OA can bias estimates of the value of relative water content, as has been shown in wheat and barley (Boyer et al., 2008).

OA has been implicated in sustaining yield under conditions of water deficit in oilseed *Brassica* species (Kumar and Singh, 1998), chickpea (Basu et al., 2007), cotton (Saranga et al., 2001), rice (Babu et al., 1999; Jongdee et al., 2002; Praba et al., 2009), sorghum (Tangpremsri et al., 1995), maize (Chimenti et al., 2006), tef (Ayele et al., 2001), barley (Gonzalez et al., 2008), and wheat (Ali et al., 1999; Blum et al., 1999; Salem et al., 2007; Ehdaie et al., 2008; Fan et al., 2008; Izanloo et al., 2008). Yet the value of OA as a desirable selection target from a breeding standpoint has been questioned (Munns, 1988; Palta et al., 2007), based on the notion that drought-tolerant genotypes endowed with a higher capacity to adjust osmotically are likely to be characterized by slow growth, and hence biomass production, due to the metabolic requirements of osmolyte biosynthesis. Under conditions of severe dehydration, a higher capacity to accumulate osmolytes may help plants withstand a prolonged drought spell and undergo a more prompt and complete recovery upon rehydration. Even though, the interpretation of osmotic relations in genetically engineered plants can be cumbersome (Blum et al., 1996), transformation experiments have shed light on the mechanisms by which plants may benefit from an altered capacity to accumulate osmolytes (Umezawa et al., 2006). Similarly to other drought-adaptive traits, the trade-off between the metabolic requirements of OA and the potential benefits for the crop varies on a case-by-case basis as a function of the crop, and the dynamics and severity of the drought episodes.

### Chlorophyll concentration, stay-green, and delayed leaf senescence

A well-sustained source capacity is a key factor to maximize yield potential during both vegetative and reproductive phases, particularly under source-limiting conditions that commonly characterize drought-stressed crops. Therefore, delaying leaf senescence maintains transpiration and increases cumulative photosynthesis over the crop life cycle (Borrell et al., 2001; Jiang et al., 2004; Vadez et al., 2011). This is a strategy that is adequate for soils with appreciable water reserves but may otherwise cause severe stress at the end of the growth season due to increased transpiration.

The traits that have been monitored most frequently to obtain indirect estimates of photosynthetic potential are chlorophyll concentration, stay-green and delayed senescence, all of which are interconnected (Tuinstra et al., 1998; Thomas and Howarth, 2000; Shukla et al., 2004). In US Corn Belt maize, stay-green has improved significantly and steadily during the past six decades of breeding, particularly under favorable conditions (Duvick, 2005). Additionally, stay-green traits in maize correlate closely to GY, and multiple intervals of stay-green QTLs overlap with yield QTLs (Zheng et al., 2009). Although, stay-green in maize seems more likely to be related to nitrogen use, in sorghum it has been related to maintenance of a more favorable water status as related to root features (Gallais and Hirel, 2004; Blum, 2006; Mace et al., 2012). In sorghum, four major QTLs that control stay-green and GY have been identified (Harris et al., 2007) and near isogenic lines (NILs) for these QTLs have been derived, providing an opportunity for a detailed analysis of stay-green physiology and positional cloning of the underlying genes (Vadez et al., 2011).

### Remobilization of water-soluble carbohydrates

Remobilization of WSC from the stem and leaves can mitigate the negative effects on grain filling caused by post-anthesis drought tolerance (Blum, 1988, 1998; Araus et al., 2002; Reynolds et al., 2007; Rebetzke et al., 2008b). QTLs for stem-reserve remobilization have been reported in bread wheat (Salem et al., 2007; Snape et al., 2007; Yang et al., 2007). Rebetzke et al. (2008b) phenotyped three wheat mapping populations for WSC concentration (WSC-C) and for WSC mass per unit area (WSC-A). Genotypes with high WSC-C were commonly shorter, flowered earlier and produced significantly fewer tillers than those of low WSC-C. This resulted in similar yields, lower final biomass, and fewer grains per m^2^, but greater dry weight partitioning to grain and kernel weight in high versus low WSC-C genotypes. In contrast, lines high for WSC-A produced more fertile tillers associated with similar or greater anthesis and maturity biomass, grain number and yield, yet similar kernel weight or size compared with genotypes with low WSC-A, thus suggesting an important role for WSC-A in assuring stable yield and grain size in wheat.

This overview of drought-adaptive traits, far from being exhaustive, indicates that genetic variability in drought tolerance and WUE can be traced to the interaction of a multitude of quantitatively inherited morphophysiological features, whose effects on yield can vary greatly both in terms of magnitude and direction according to the prevailing drought scenario and other yield constraints. Therefore, the adoption of drought-adaptive traits as selection criteria for yield should be exercised cautiously and only after acquiring a clear understanding of the factors limiting yield in the TPE. Identifying the QTLs underpinning such traits and interpreting their cause–effect relationships allow us to partially disentangle this complexity to an extent and, eventually, make it amenable to a more direct and effective manipulation for breeding purposes. In both cases, good phenotypic data are essential to success.

## Collecting good phenotypic data

Plant scientists attempting to improve resistance to drought face two contrasting and apparently irreconcilable requirements. The first is to simplify “the system” in order to facilitate elucidation of the function of the relevant loci for the target traits (i.e., the reductionist approach). The second is to evaluate the broader value of such findings in a breeding and agronomically sound context (i.e., the holistic approach), where the physiology, epistatic interactions and pleiotropic effects of complex traits inevitably limit and blur the identification of the main factors leading to specific phenotypes (e.g., drought-resistant versus drought-susceptible). In a way, the reductionist approach is like trying to understand the subject of an entire puzzle when only a few pieces are available. On the other hand, the holistic approach selecting, for example, for yield *per se* will provide a complete picture of the puzzle (i.e., the phenotype). However, it will often not allow us to tease the puzzle apart to the extent that we would need to apply targeted approaches such as MAS and/or genetic engineering, because of our incomplete understanding of the number and function of the single pieces such are the QTLs for yield. Valuable opportunities to begin to reconcile this conundrum are provided by bioinformatics (Sawkins et al., 2004) and modeling (Hammer et al., 2004, 2006; Cooper et al., 2009; Tardieu and Tuberosa, 2010; Sinclair et al., 2010; Messina et al., 2011). Both modeling and high-throughput phenotyping for drought-adaptive features are at the very core of DROPS (DROught-tolerant yielding PlantS; www.drops-project.eu), an ongoing EU-funded project aiming at improving our understanding and capacity to ameliorate yield and yield stability under water-limited conditions.

Yet the objective of this review is not to dwell on the merits and pitfall of the reductionist and holistic approaches (see also Passioura, 2010). Rather, it seeks to introduce and discuss a number of major issues on phenotyping that are relevant for both approaches. These issues should be considered seriously in planning and managing experiments under drought conditions, collecting and analyzing the data and, eventually, in interpreting the results properly.

Given the myriad of factors that can influence the quality of phenotypic data, this review only addresses the most important ones. Although, it is possible to define general rules, each experiment has its own “phenotyping story” and the results should be dealt with and interpreted accordingly. What follows is equally relevant for the improvement of crop performance under water-limited conditions and, more generally, for experiments in the field or under controlled conditions aimed at dissecting the physiological and genetic basis of crop adaptation to water-limited conditions. However, given the importance of field evaluation for breeding purposes, phenotyping under field conditions is emphasized.

### What does “good phenotyping” mean?

Good phenotyping is pivotal for reducing the genotype–phenotype gap, especially for quantitative traits, which are the major determinants of drought resistance. Keeping a good record of meteorological parameters (rainfall, temperatures, wind, evapotranspiration, light intensity, etc.) allows for more meaningful interpretation of the results and identification of the environmental factors limiting yield (Sadras, 2002). Equally important, though often neglected or ignored, are the physical-chemical properties of the soil, particularly those influencing the water balance of the crop under decreasing moisture conditions (Cairns et al., 2011).

The basic attributes of good phenotyping carried out with appropriate genetic materials are accuracy and precision of measurements, coupled with relevant experimental conditions that are representative of the TPE. Accuracy involves the degree of closeness of a measured or calculated quantity to its actual (true) value. Accuracy is closely related to precision, also termed reproducibility or repeatability, the degree to which further measurements or calculations show the same or similar results. For a number of traits such as stomatal conductance, flow of xylem sap, etc., measured with mechanical or electronic devices, accuracy and precision in measurements require calibration of the instrument prior to data collection. Failure to so do will produce biased results with a difference between the mean of the measurements and the true reference value. A further complexity of phenotyping a large number of genotypes (e.g., a mapping population or an association mapping panel) for drought-adaptive features is exemplified by those traits such as stomatal conductance and tissue water potential, the value of which can vary considerably within a rather short timeframe due to changing environmental conditions.

An important distinction should be made between experiments aimed at (1) collecting data useful to dissect the genetic basis of target traits or (2) breeding activities for the release of improved cultivars. In both cases, an adequate choice of materials will be essential for successfully meeting the desired objectives. A notable case that clearly underscores the importance of good phenotyping is provided by QTL cloning (Salvi and Tuberosa, 2007). In this respect, the ideal scenario is when the alternative QTL alleles can be unequivocally scored phenotypically and the trait itself is mapped as one of the markers.

### Phenotyping is king and heritability is queen

Good phenotyping means not only the collection of accurate data to minimize the experimental “noise” introduced by uncontrolled environmental and experimental variability, but also the collection of data that are relevant and meaningful from a biological and agronomic standpoint, under the conditions prevailing in farmers' fields within the TPE. Although, hundreds of accurate studies reporting thousands of drought-responsive genes and QTLs can be found in the literature, the relevance of these data to “real” field conditions is often marginal and even questionable; only seldom has it been appropriately addressed and discussed. In the early stages following their development, evaluation of transgenic materials is limited to experiments carried out in greenhouses, a condition that underlines the importance to mimic as close as possible the drought stress conditions in fields (Saint Pierre et al., 2012).

Collecting accurate phenotypic data that are relevant to the TPE has always been a major challenge for the improvement of quantitative traits. The success of this endeavor is intimately connected with the heritability of the trait, namely the portion of the phenotypic variability accounted for by additive genetic effects that can be inherited through sexually propagated generations (Falconer, 1981). Trait heritability varies greatly (from 0 to 1) according to: (1) the genetic makeup of the materials under investigation; (2) the environmental conditions under which such materials are grown and evaluated; and (3) the accuracy and precision of the phenotypic data. With only a few notable exceptions (e.g., flowering time and carbon-isotope discrimination), most of the traits determining the performance of crops under drought conditions usually have low (0.3–0.4) or, at best, intermediate (0.4–0.7) heritability. This impairs our capacity to dissect their genetic basis properly and, more importantly, reduces the effectiveness of phenotypic selection (Falconer, 1981). Despite this, careful evaluation and appropriate management of the experimental factors that lower the heritability of traits, coupled with a wise choice of the genetic material, can provide effective ways to increase heritability, and hence the response to phenotypic selection.

Once a sound association has been established between a marker and a locus affecting a target trait, the problems encountered in the conventional selection of quantitative traits, particularly the lowly-heritable ones, can been partially overcome through the use of markers linked to QTLs for the target trait. This enables individuals to be scored based on their genetic make-up rather than their phenotypic features (Peleman and Van der Voort, 2003; Langridge, 2005). Paradoxically, the probability of identifying the relevant chromosomal regions and accurately estimating their effects relies on good phenotyping of the genetic materials originally used to establish the phenotype–genotype associations. In other words, the effectiveness of marker-based approaches intimately depends on how well and how accurately the target trait has been assessed phenotypically in mapping populations. In fact, a low heritability impairs the probability of detecting the presence of QTLs (Bernardo, 2004), thereby increasing Type II errors (i.e., false negatives). An accurate and relevant phenotyping is of even greater importance when applying genome-wide selection, an approach that disregards QTL identification and relies on the molecular profiling and accurate phenotyping of each progeny (Bernardo and Yu, 2007; Bernardo, 2008; Heffner et al., 2009).

### Experimental design, dedicated software, and statistical approaches

It is widely recognized that a substantial part of the increased efficiency of modern breeding is due to the accurate phenotyping of large numbers of plots, this scale-up being made possible by more sophisticated and high-throughput experimental machinery as well as the streamlining and automation of tedious manual operations. Thus, the labeling of a large number of plots and samples, data collection and storage, and keeping track of pedigrees, etc., are now facilitated by the use of electronics (e.g., bar-coding) and dedicated software (e.g., spreadsheets, databases, etc.). Additionally, the effectiveness of field experiments and the management and interpretation of phenotypic data can be enhanced greatly through the utilization of the most appropriate experimental designs to allow for better control of within-replicate variability and to reduce or remove spatial trends. Equally important are statistical approaches to analyzing the data, particularly for investigating the effects of GEI (van Eeuwijk et al., 2005; van Eeuwijk, 2006; Malosetti et al., 2008; Mathews et al., 2008; Messmer et al., 2009) and epistasis (Gao and Zhu, 2007; Jannink, 2007). Coping with the temporal variability of drought-adaptive features can be dealt with through in-depth analysis of QTL-by-environment interactions (van Eeuwijk et al., 2005; Vargas et al., 2006; Burgueno et al., 2008) or by identifying intrinsic characteristics of each genotype relating to its interaction with particular environmental conditions, which requires the development of models able to identify these variables and to simulate the behavior of genotypes in a broad range of environments (Tardieu, 2003; Yin et al., 2003; Reymond et al., 2004; Cooper et al., 2009; Sinclair et al., 2010).

A number of studies have shown the importance of epistasis in determining the genetic architecture of yield and other quantitative traits (Li et al., 2003b; Maccaferri et al., 2008; Zhao et al., 2008; Frascaroli et al., 2009; Messmer et al., 2009; Ravi et al., 2011). However, mapping two-way epistatic interactions requires adequately large mapping population, and detecting higher order epistasis is practically out of reach. Once different sets of NILs become available for loci that are known to interact epistatically, it will be possible to produce different combinations at will for further testing and characterization of the effects of such epistatic interactions.

## Monitoring plant–soil water relations

A sound interpretation of the results of an experiment conducted under conditions of water shortage requires a good characterization of the soil–plant–atmosphere continuum (SPAC), which, in turn, relies on accurate monitoring of the water status of both soil and plant. From an experimental standpoint, an important issue is to what extent genotypic differences in drought-adaptive traits measured in phenotyping platforms at different water regimes reflect genotype performance across watering regimes under field conditions. Along this line, encouraging results have recently been reported in maize (Chapuis et al., 2012).

Regrettably, a unique means of measuring water status that can be applied in all possible situations is not available. Choosing the most appropriate method depends on the objective being pursued, such as understanding drought-adaptive mechanisms, selecting for drought resistance, investigating water movements, or managing irrigation treatments (Boyer, 1995; Kirkham, 2004; Jones, 2007). At the plant level, greater emphasis has traditionally been devoted to water potential rather than sustained turgor, the primary reason for sustained function under drought (Blum, 2006, 2009). Hence, examples of sustained function at low water status as the main reason for drought tolerance are comparatively few. Maintenance of high leaf water potential and turgor under dry conditions indicates dehydration avoidance (Blum, 1988; Ludlow and Muchow, 1990). Similarly, the relative water content of the leaf also provides important information on the water status of the plant, offering the advantage of collecting a high number of samples in a short time (Sanguineti et al., 1999), an important prerequisite for QTL studies trying to link variation in physiological parameters to variation in yield. The precautions to be adopted for measuring relative water content have been discussed by Blum^2^. Although, all components of leaf water relations change during the day as irradiance and temperatures vary, the change is small for about 2 h at and after solar noon. Therefore, this is an appropriate time window for investigating leaf water relations in a large number of genotypes^2^.

It is equally important to monitor changes in soil moisture, preferably at different depth of the rhizosphere, during the growth and reproductive cycle of the crop. Root water uptake is one of the pivotal processes within the SPAC. While the gravimetric method (i.e., weighing samples of soil columns before and after oven drying) provides accurate, albeit time-consuming, measurement of soil moisture, other methods such as the neutron probe, the capacity method and the “I-sensor” allow for quicker and less labor-intensive measurement (Nagy et al., 2008; Cayci et al., 2009).

During recent decades, progress in microelectronics has allowed the development of several dielectric-based soil water monitoring techniques, namely time-domain reflectometry (TDR), and single and multisensor capacitance probe (SCP/MCP) systems (Fares and Polyakov, 2006; Vereecken et al., 2008). These techniques have greatly simplified the real-time determination of water content on a fine spatial and temporal scale. Because of their relatively low cost and ease of operation, MCP systems have met widespread acceptance as a means of closely monitoring soil moisture by collecting high-resolution soil-water content data in the rhizosphere. Despite their success, MCP systems have shown some temperature and salinity effects in different soil types, suggesting that further research is needed to eliminate such effects for these capacitance systems to take their place as leading soil water monitoring sensors.

TDR has been one of the most widely used techniques to determine soil volumetric water content thanks to its high precision, non-ionizing radiation and low influence of soil salinity, bulk density and texture (Noborio, 2001). However, compared to the neutron probe, most of the TDR equipment available does not allow detailed measurement along the soil profile. Also, the use of conventional TDR probes requires drilling holes or opening trenches in the soil to install the probes, limiting the number of points measured in the soil profile (Manieri et al., 2007). More recently, two-dimensional geoelectrical tomography has been used for monitoring soil-water redistribution due to water uptake by lupin roots (Werban et al., 2008). The resulting average water content from two-dimensional geoelectrical tomography agreed well with the values determined by the TDR measurements model.

## What severity of water shortage?

Unlike yield under conditions of severe drought stress (>70% reduction from yield under well-watered conditions) yield under more moderate water shortage (up to approximately 50% reduction) reflects more closely yield potential under favorable conditions (Blum, 2006). Therefore, drought resistance *per se* is expected to play a progressively more important role than yield potential as the severity of drought escalates, with genotype ranking for yield changing considerably once the mean yield falls below 20–30% of yield potential (Blum, 2006) as a result of water scarcity. Consequently, germplasm evaluation in areas where drought severity fluctuates widely should preferably be carried out under well-watered conditions and at different levels of drought stress (e.g., intermediate and severe). In maize, this approach has been adopted to identify QTLs for yield across a broad range of water availability (Malosetti et al., 2008; Messmer et al., 2009) and to develop superior hybrids in sub-Saharan Africa (Bänziger et al., 2006).

Retrospective studies conducted with an historical series of maize hybrids showed that screening in multiple sites at high plant densities provides substantial yield gains across a broad range of environments, although, rates of gain in well-watered conditions are more than twice as high as those in water-stressed environments (Duvick, 2005; Campos et al., 2006). In wheat, four decades of breeding at CIMMYT have clearly indicated the importance of selecting and managing key environments differing in their yield potential to identify the best performing genotypes across a broad range of environments. The so-called “shuttle breeding” which was instrumental for the success of the Green Revolution (Borlaug and Dowswell, 2005), remains a key factor in developing more broadly adapted cultivars (Ortiz et al., 2007b; Trethowan and Crossa, 2007). Recently, a QTL with a major and consistent effect on GY in multiple elite genetic backgrounds under both water-stressed and non-stressed conditions has been described (Vikram et al., 2011). Consistency of the QTL effect across different genetic backgrounds makes it a suitable candidate for use in marker-assisted breeding.

## Phenotyping in the field

Assuming that both the type and the number of treatments (genotypes, irrigation volumes, etc.) to be evaluated are adequate for the specific objectives of each experiment, the following general factors should be evaluated carefully to ensure the collection of meaningful phenotypic data in field experiments conducted under water-limited conditions:

- Experimental design
- Heterogeneity of experimental conditions between and within experimental units
- Size of the experimental unit and number of replicates
- Number of sampled plants within each experimental unit
- Genotype-by-environment-by-management interaction.

The relative impact of each factor on the quality of the phenotypic data to be collected will vary greatly according to each experiment. As an example, an excessive heterogeneity in soil characteristics (depth, moisture, pH, etc.), and/or compaction among field plots will inevitably increase the experimental error and will jeopardize an accurate evaluation of yield. Mapping the soil in experimental nurseries for environmental factors that decrease phenotypic accuracy (Cairns et al., 2004, 2011; Rossel et al., 2006; Patzold et al., 2008) and adopting suitable experimental designs can partially mitigate the negative effects of high soil heterogeneity.

For experimental activities carried out under drought conditions, the additional factors discussed below should receive due attention when planning and conducting the experiments.

### Variation in phenology

In environments where escape is the predominant cause of drought resistance, the presence of large differences in flowering time among genotypes will inevitably bias the interpretation of the influence of drought-adaptive traits on yield under drought conditions (Soltani and Sinclair, 2012). Likewise, the presence of large differences in plant height and/or root mass among the progeny of a mapping population or accessions of a panel suitable for association mapping studies, may lead to an overestimate of QTL effects owing to competition between neighboring plots, especially when their surface area is small. These QTL effects will most likely decrease once phenotypic evaluation has been carried out with more phenologically homogeneous materials. Surprisingly, this issue has not yet been addressed with dedicated experiments.

### Interactions with other stresses

Obtaining an accurate estimate of drought resistance *per se* implies the absence of other biotic or abiotic stress agents that influence plant growth and function. Typical case scenarios are those involving factors that cause mechanical damage to roots (e.g., nematodes, root-worms, etc.), impair root growth (e.g., soil acidity, boron toxicity, salinity, etc.), and/or reduce water availability to the crop (e.g., presence of weeds), and source capacity (e.g., foliar diseases, insect damage to the canopy, etc.). When one or more of the above-mentioned constraints affects the experimental plots, genetic variability among the progeny in resistance to these stress agents will inevitably bias an accurate evaluation of drought resistance. Likewise, important and more subtle interactions may occur when the effects of water deficit are evaluated in the presence of other abiotic stress factors (e.g., high temperatures, high ozone, low nutrients, etc.) that hasten leaf senescence and/or enhance the role of specific adaptive mechanisms, such as the relocation of stem WSC in cereals, that normally play a less predominant role in determining yield.

Nevertheless, it should be noted that drought hardly ever occurs in the absence of other stress factors (Sadras, 2002; Sinclair et al., 2007). An example of this is provided by the conditions of terminal drought stress frequently concomitant to high temperatures that wheat and rice experience during grain filling (Pinto et al., 2010; Jagadish et al., 2011; Lopes et al., 2012; Yang et al., 2012). A partial solution to this problem, at least for traits other than GY and its components, which are best evaluated under field testing, is to collect phenotypic data from plants grown in controlled facilities (greenhouse, growth chamber etc.). This will allow for an accurate control of the main environmental parameters—temperature, air humidity, light, etc.,—governing water flow in the SPAC, and hence the water balance. This is particularly important for omics-profiling studies where even small fluctuations in environmental conditions can substantially alter gene expression. On a broader scale, environmental characterization can be improved through the use of geographic information systems (GIS) for crop monitoring (Kahinda et al., 2008), for water balance models (Reshmidevi et al., 2008) and for their combination.

### Managing the dynamics and intensity of drought episodes

The ability to control the timing, frequency and intensity of drought episodes is a key factor in mimicking the environmental conditions prevailing in the TPE and, consequently, in successfully selecting for improved drought resistance. To this end, an increasing number of public and private breeding programmes have conducted field trials in locations characterized by very low rainfall during the growing season, a condition under which the dynamics and intensity of drought episodes can be tightly controlled through the frequency and volume of irrigation treatments. Trials in dry sites also offer the distinct advantage of a lower incidence of biotic constraints which, if unaccounted for, can bias the evaluation of the role of other traits and corresponding QTLs in the adaptive response to moisture-limited conditions.

The option of field testing in dry areas is not always available to many of those engaged in drought-related experiments. Therefore, rainout shelters offer the possibility of investigating the adaptive response of crops to a desired level of drought stress, avoiding the vagaries of unpredictable rainfall patterns. There are basically two types of rainout shelter: static and moveable. Further details on the merits and pitfalls of these devices are provided by Blum^2^. Major drawbacks to the use of rainout shelters are high construction and operating costs, particularly for the movable type, as well as the usually rather limited area protected by a shelter which, in turn, limits the number and size of experimental plots that can be tested. This is a significant problem when dealing with large mapping populations or panels of accessions suitable for association mapping studies.

### Influence of the growth stage

An important aspect for phenotyping traits in the most relevant way from a breeding point of view is the identification of the critical stage at which variability in the target traits plays a more prevalent role in final performance. This is the stage at which the correlation between the trait and final yield is highest, and thus becomes more diagnostic. For example, in maize some biochemical factors, such as the concentration of sucrose in the placental-chalazal area of the kernel, exert a particularly strong and timely effect on reproductive fertility around flowering but not a week earlier or later (Boyer and Westgate, 2004; McLaughlin and Boyer, 2004). Similarly, genetically-based differences in the concentration of ABA in leaves of field-grown maize have been shown to peak around the time of flowering or shortly after (Landi et al., 1995; Pekic et al., 1995). Due consideration should also be given to fluctuations in the heritability of target traits exhibited during the growth cycle (see below).

A critical factor in improving the relevance of infrared thermography to measure canopy temperature is the timing of the measurements of temperature differences between treatments. Under field conditions, even well-watered healthy plants may shut their stomata before solar noon, especially under conditions of high evapotranspirative demand. This is particularly relevant when different genotypes are evaluated for their capacity to exploit an avoidance strategy. In this case, the timing of the measurements to allow good discrimination among genotypes needs to be determined for specific conditions and may need considerable readjustment during subsequent samplings as the water stress progresses during the day. An additional factor to be considered when measuring canopy temperature is the effect of leaf wilting, folding or rolling under stress (Leinonen et al., 2006; Grant et al., 2007). For instance, plant canopy architecture will influence leaf temperature not only through the angle of leaves to the light source, but also through the degree of self-shading in the canopy (Zheng et al., 2008). To a certain extent, the influence of self-shading can be reduced if the most suitable view angle is used, although, different opinions have been expressed in this regard (Grant et al., 2006).

When phenotyping occurs at flowering or shortly after, additional bias is introduced if the tested genotypes differ considerably in flowering time and/or maturity. In such cases, phenotyping all accessions on the same date will provide data collected from plants at different physiological stages, a circumstance that could introduce significant bias in the interpretation of cause–effect relationships between traits and yield. A partial solution is to sow the accessions on two or three dates based on the maturity group (e.g., early and late). Clearly, this procedure will increase the cost of the trial.

### Timing of measurement and sample collection

For morphophysiological traits that fluctuate widely during the circadian cycle (e.g., water status, ABA content, stomatal conductance, leaf rolling, leaf temperature, etc.) choosing the most appropriate time for measurement and/or sample collection is very critical. Additionally, measurement of traits that are time-consuming to record (e.g., stomatal conductance) in a large number of plants introduces a covariate effect proportional to the duration of data or sample collection. In this respect, remote sensing holds great potential to minimize or eliminate altogether effects on trait expression due to the circadian rhythm and corresponding changes in environmental factors.

CTD is a notable indicator of the amount of water extracted from the soil and lost through foliar evapotranspiration into the atmosphere. Therefore, this trait provides an indirect estimate of root architecture (size and depth) and functionality (e.g., permeability to water as a function of aquaporines, etc.) in accessing soil moisture, and can be used as a fast, inexpensive screening of root features (Reynolds et al., 2009). However, to be diagnostic, canopy temperature should be measured under conditions of high evapotranspirative demand and in absence of wind (Blum, 1988), since even a slight breeze can alter the level of evapotranspiration instantaneously and, consequently, alter the leaf temperature. Balota et al. (2007) have investigated the effects of the timing of measuring CTD on breeding selections of wheat in relation to growth stage, time of day and weather. Although, under dry conditions long-term mean CTD at noon and yield were found to be correlated in two growing seasons, the relation of short-term CTD readings to GY was highly variable (Balota et al., 2007). Poor correlation was associated with days of low solar irradiance, high wind speed and rain events. Interestingly, genotype effects on CTD were detected for all hours of day and night. Genotype-by-hour interaction was non-significant at night, suggesting that night-time measurements may provide more stable conditions for CTD comparison among genotypes.

## Phenotyping in controlled environment facilities

Although, GY and its components are best phenotyped in field trials, measuring secondary traits in plants grown in controlled environment facilities (e.g., greenhouse, growth chamber, etc.) takes advantage of an accurate control of the main environmental parameters of moisture stress, air humidity, temperature, light, etc., that vary greatly in field experiments. However, the conditions under which plants are grown should be relevant to the conditions prevailing in the field (Izanloo et al., 2008). When the materials under test differ in flowering time, the use of plants grown under controlled conditions facilitates the collection of phenotypic data and samples at the same growth stage and under similar conditions. Additionally, a tight control of growing conditions allows for more accurate assessment of the constitutive capacity of different genotypes to accumulate drought-adaptive compounds in response to a given level of water deficit. For example, the accumulation of osmolytes and/or ABA is highly influenced by water status, which can vary considerably among genotypes tested in the field under similar water regimes (Tuberosa et al., 1994; Rauf et al., 2009).

More uniform conditions in terms of water status can be achieved through exposing plants to a solution with a known concentration of polyethylene-glycol (PEG). This approach can be of particular interest as a way of exposing different genotypes to a given level of dehydration (Sanguineti et al., 2006; Verslues et al., 2006; Texeira et al., 2008; Ruta et al., 2010). Unlike in field conditions where different genotypes are likely to experience different stress intensities, plants grown in a PEG solution are exposed to predetermined and rather uniform water stress, a condition that facilitates a more correct interpretation of the cause–effect relationships of the association between traits. However, the use of PEG requires good aeration of the solution to avoid hypoxia and verification of the absence of possible contaminants. Additionally, plants absorb PEG, particularly when it is of a low molecular weight (<6000), which can alter the hydraulic properties of the leaf^2^. Therefore, great caution should be adopted in taking results obtained under such highly artificial conditions and extrapolating them to field conditions.

In most circumstances, the collection of phenotypic data in experimental conditions that are remote from those prevailing in the field may lead to biased and potentially misleading conclusions. At the molecular level, an interesting example is provided by transcriptomics studies (Atienza et al., 2004; Rensink, 2005) wherein plants or plant parts such as detached leaves undergo high-intensity stress treatments in a rather short time, i.e., “shock-like” treatments. These conditions preclude the identification of long-term responses in gene expression that play a more predominant role in adaptation to field aridity (Passioura, 2010). In barley, changes in gene expression were monitored in leaves of plants grown in soil and subjected to slow-drying conditions for 7 and 11 days (7d-WS and 11d-WS, respectively) with the changes obtained under “shock-like” conditions imposed with a 6 h dehydration treatment (Talamè et al., 2007). Among all transcripts that showed a significant change in regulation in at least one of the conditions tested, 57% were exclusively affected in the dehydration shock treatment, 6% at 7d-WS and 14% at 11d-WS. Irrespective of the low percentage of transcripts (10%) with similar expression changes between shock- and slow-stress treatments, a portion of these transcripts shared a common expression trend under the different drought treatment conditions, as evidenced by low correlations between the fast-occurring and the 7d-WS and 11d-WS treatments (*r* = 0.32 and 0.41, respectively). From a practical standpoint, these results suggest that the information obtained under artificial conditions of water deficit induced over a very short period of time (e.g., a few hours) should be treated very cautiously when used to identify candidate genes for QTLs of field-related traits with a drought-adaptive role.

## Harnessing phenotypic variability

A number of options are available to utilize the information collected through phenotypic evaluation of germplasm resources (Gur and Zamir, 2004; Dreccer et al., 2007; Reynolds et al., 2007; Richards et al., 2007; Ortiz et al., 2008; Bernardo, 2009; Di Bianco et al., 2011; Tuberosa et al., 2011a). A well-informed choice of the parental lines based on a thorough phenotypic characterization of the main traits imparting drought resistance allows for the creation of new populations where segregants that combine drought-adaptive and other desirable features of parental lines can be identified and selected (Reynolds et al., 2005). This so-called “strategic crossing” has been deployed extensively and successfully at CIMMYT, as shown by the fact that several newly released improved wheat accessions have been selected from crosses between parental lines chosen based on their morphophysiological features (Reynolds et al., 2005, 2011; Ortiz et al., 2007b).

An effective breeding programme relies on the availability of sufficient genetic variability for the target traits. Under this aspect, landraces and wild accessions provide valuable opportunities to enhance the variability for drought-adaptive features and, eventually, yield (Moncada et al., 2001; Talamè et al., 2004; Tan et al., 2008). There is rapidly growing interest in wild relatives of crops and landraces as sources of agronomically superior alleles among those that were left behind by the domestication bottleneck and modern agriculture (Tanksley and McCouch, 1997; Lippman et al., 2007; Reynolds et al., 2007; Feuillet et al., 2008). Advanced-backcross QTL analysis (ABQA) and introgression libraries (ILs) allow for proper and effective dissection of the phenotypic variability contributed by non-commercially viable parental lines (Talamè et al., 2004; Tan et al., 2008; Salvi et al., 2011). Once a desirable QTL feature contributed by unadapted materials tested under drought conditions has been identified, the main issue is to evaluate to what extent the introgression of the target segment in elite materials might cause a yield penalty under favorable conditions. Regarding target traits, landraces and wild relatives have been screened most commonly to identify accessions with an outstanding expression of secondary traits such as root mass, OA, leaf anatomy, etc., thought to play an important role in conferring resistance to drought (Grando and Ceccarelli, 1995; Peleg et al., 2007, 2008).

## Toward high-throughput phenomics

High-throughput phenotyping helps standardize and improve the collection of phenotypic data and facilitates the creation of repository databases useful for QTL meta-analyses (Lippman et al., 2007; Welcker et al., 2011). Unlike a decade ago, our present capacity to conduct high-throughput molecular profiling far outweighs our capacity to collect reliable phenotypic data (Sinclair and Purcell, 2005). The best example is provided by the burst in single nucleotide polymorphism (SNP) discovery and profiling in a number of crops (Rostoks et al., 2005; Kota et al., 2008; Ganal et al., 2009; Waugh et al., 2009; Mondini et al., 2011; Rafalski, 2011; Trebbi et al., 2011). Nevertheless, the past years have witnessed a growing awareness of the need for increasingly integrated, multidisciplinary and field-oriented research in order to mitigate the negative effects of water shortage (Edmeades et al., 2004; Tuberosa et al., 2007a).

High-throughput phenotyping of plants in pots allows for tight control of the water shortage imposed on different genotypes and of the homogeneity of the severity of stress, a condition that is seldom achieved under field conditions, particularly when the genotypes under test differ in phenology and/or biomass. However, a number of distinct limitations characterize pot experiments and should be carefully considered and managed to obtain meaningful results relevant to field conditions (Passioura, 2006).

Phenotyping under controlled conditions is relatively straightforward when scoring traits in a binary fashion, such as for photoperiod sensitivity, and when environmental conditions do not have much effect on the target trait or are easily defined (e.g., light versus darkness). However, it quickly becomes more complex when the target traits are quantitatively assessed, as in the case of growth, and when environmental conditions that vary during the day (e.g., temperature, light intensity, soil water status, etc.) influence the target trait (e.g., the rate of leaf elongation). In this case, the phenotype is rather dynamic and better defined by a series of response curves to environmental stimuli (Tardieu et al., 2003, 2005; Hammer et al., 2004; Tardieu, 2012), an approach that is very time-consuming and requires a tight control of environmental conditions.

Hence, it is important to: (1) measure the physical variable/s (e.g., pot weight, soil moisture etc.) that quantify the level of water stress; and (2) add a precise amount of water to each pot. High-throughput phenotyping platforms allow for the automation of these procedures that have already been adopted by a number of private companies and large public institutions to streamline and standardize the collection of highly accurate phenotypic data in glasshouse-grown plants (Granier et al., 2006; Rajendran et al., 2009). State-of-the art technology including imaging, robotic and computing equipment, allows for the continuous phenotypic measurement of thousands of plants automatically and non-destructively^3^. Regrettably, the installation and operating cost of these platforms is very high.

For certain traits, the high-throughput collection of phenotypic features can be streamlined by the use of digital imaging and measurement of canopy features by means of near-infrared spectroscopy and spectral reflectance, as discussed below.

### Digital imaging

Digital image analysis provides an inexpensive and rapid way of precisely measuring plant features whose measurement would otherwise require a great deal of time. A notable example is provided by the measurement of canopy features (Marti et al., 2007; Campillo et al., 2008; Elsayed et al., 2011; Winterhalter et al., 2011b; Fiorani et al., 2012). Digital images offer a series of advantages over other methods of light interception estimation, including the possibility of directly processing images by computer. Video image analysis allows for a dynamic, inexpensive and non-destructive assessment of canopy features and crop growth (Beverly, 1996; Campillo et al., 2008; Cairns et al., 2011; Elsayed et al., 2011; White et al., 2012). Digital imaging is equally valuable for measuring root characteristics in experiments that are often constrained by the lack of suitable methods for continuous, non-destructive measurements (Himmelbauer et al., 2004; Blouin et al., 2007). Additionally, digital image analysis (Kimura et al., 1999; Armengaud et al., 2009) allows for accurate analysis at higher resolution scales, an important prerequisite to investigate the kinetics of the processes regulating root growth. In this respect, a non-invasive technique, based on digital image sequence processing, has been applied for quantifying highly resolved spatio-temporal processes within the root growth zone in the model plant Arabidopsis (Chavarria-Krauser et al., 2008; Iyer-Pascuzzi et al., 2010).

### Near-infrared spectroscopy and spectral reflectance

Remote sensing via near-infrared spectroscopy and spectral reflectance of plant canopies are promising components of high-throughput phenotyping platforms (Montes et al., 2007) and provide interesting opportunities for collecting integrative traits with high temporal resolution (Gutierrez et al., 2010). Spectral reflectance in the visible and near-infrared regions of the electromagnetic spectrum is collected from the canopy of the crop by sensors that can be mounted on tractors (Montes et al., 2007) or using digital cameras mounted on hand-held devices (Casadesus et al., 2007). Remote sensing has advanced our understanding of the changes in leaf reflectance and leaf emittance according to species, leaf thickness, canopy shape, leaf age, nutrient status and, importantly, water status (Hatfield et al., 2008). Based on this information, various vegetative indices for crop canopies have been formulated to quantify agronomic parameters (e.g., leaf area, crop cover, biomass, yield, etc.). Retrieving meaningful information from the plot spectra relies on the use of calibration models for prediction of the phenotypic values. Under well-managed experimental conditions, spectral reflectance has been used to monitor plant photosynthetic pigment composition, water status assessment and the early detection of abiotic stress (Babar et al., 2006, 2007; Guo et al., 2008; Gray et al., 2010).

## Simulating virtual phenotypes

As we inch our way forward to unravel gene functions in a piecemeal fashion (i.e., gene-by-gene) and try to understand how these functions ultimately affect the phenotype, there is a growing interest in models that allow us to simulate virtual phenotypes deriving from all possible combinations of different factors—alleles, environmental variables, etc. In a way, modeling represents a step toward a more comprehensive systems biology approach (Dingkuhn et al., 2005; Yin and Struik, 2008; Tardieu and Tuberosa, 2010) aimed at predicting phenotypic performance of an otherwise intractably large number of treatments, such as the genotypes obtained by combining different gene/QTL alleles, irrigation volumes and frequency, temperatures, etc., (Hoogenboom et al., 2004; Cooper et al., 2007; Heinemann et al., 2008; Letort et al., 2008; Sinclair et al., 2010).

The assumption is that gene networks are regulated in a coordinated way to allow plants to react predictably to a range of environmental conditions (Sadok et al., 2007; Chenu et al., 2008; Jansen et al., 2009; Chapuis et al., 2012). Crucial to the success of this approach is the possibility of monitoring the phenotype of each accession in a precise and rapid way for the target trait (e.g., leaf elongation) in response to closely controlled environmental variables such as temperature, evaporative demand, soil water status, etc. Clearly, this kind of study is best conducted under controlled conditions. In maize, the QTL parameters of these responses were calculated for lines of mapping populations and were then analyzed genetically (Reymond et al., 2003; Welcker et al., 2007), allowing simulation of leaf growth in novel inbred lines as defined by their QTL alleles (Sadok et al., 2007). Therefore, this approach allows for the identification of QTLs of plant responses that, in principle, should not include a GEI. It theoretically allows prediction of the performance of any “virtual genotype” with a given combination of alleles in any climatic scenario. This possibility opens up a promising avenue, but is limited at present to very simple traits and genetic systems.

More integrative models simulate crop development as a function of environmental conditions. Consequently, they allow for the evaluation of the effects of individual traits on the seasonal dynamic of water use and carbon assimilation of crops (Chapman et al., 2003; Yin et al., 2004). However, their algorithms remain relatively crude, so the effects of genes or QTLs cannot usually be simulated at the crop level except for constitutive traits such as phenology (Chapman et al., 2003; Yin et al., 2005), for binary traits related to environmental triggers, such as flowering response to photoperiod (Hoogenboom et al., 2004) or when QTL models at the organ level can be combined with crop models (Chenu et al., 2008; Tardieu and Tuberosa, 2010). Their main function until now has been to evaluate whether a given trait will have a positive effect over a long series of climatic scenarios. For instance, Hammer et al. (2005) simulated the effect of stay-green, a trait considered as conferring drought tolerance, across 547 location-season combinations. As expected, this trait had a positive effect under mid-season or terminal stress, but a negative effect under severe terminal stress.

A factor that affects the prediction capacity of modeling is the unaccounted complications caused by non-linear effects associated with genes acting in networks when selection is conducted on a population of individuals segregating for the genes contributing to the network (Peccoud et al., 2004). Notwithstanding the promising features of modeling, an accurate prediction across genotypes still remains a difficult undertaking.

## Conclusive remarks and perspectives

Taking full advantage of germplasm resources and the opportunities offered by genomics approaches to improve drought resistance will require a better understanding of the physiology and genetic basis of drought-adaptive traits. Clearly, an accurate and cost-effective phenotyping will be instrumental in this respect. The utilization of techniques/approaches that allow for a precise control of the water regime (e.g., irrigated trials in dry regions, rainout shelters, etc.) and a reduction of the experimental noise coupled with the adoption of high-throughput platforms will streamline the collection of good phenotypic data while increasing the cost-effectiveness of phenotyping. This, in turn, will help to lift, at least partially, the “statistical fog” that surrounds QTLs and impairs our capacity to properly gauge their effects and predict the potential of novel combinations of QTL alleles.

However, no matter how accurate our phenotyping will be, the vast majority of the QTLs determining the measured phenotype will remain undetected. By analogy, I refer to this as the “iceberg effect.” Similar to an iceberg, where most of mass lies below the sea surface and thus is not visible, the majority of the genetic factors controlling quantitative traits will equally defy detection because their effects are simply too small to be evidenced at a statistically significant level. Therefore, notwithstanding the implementation of new crossing schemes (e.g., multiparental crosses: Blanc et al., 2006, 2008) and approaches (e.g., association mapping: Buckler et al., 2009; Lu et al., 2012; Maccaferri et al., 2011; Varshney et al., 2012) that facilitate the identification and cloning of QTLs, the targeted manipulation of yield will remain a daunting undertaking.

As compared to MAS, genome-wide selection, while bypassing QTL identification (Bernardo, 2009), relies even more so on accurate phenotyping. As the cost of genotyping and sequencing keeps dropping (Varshney et al., 2009; Feuillet et al., 2011), cost-effective phenotyping will become increasingly strategic for further dissecting drought-adaptive traits and tailoring cultivars better suited for farming under drought-prone conditions. Hopefully, the information presented in this review will help raising interest in phenotyping as well as due awareness and appreciation of its pivotal role.

### Conflict of interest statement

The author declares that the research was conducted in the absence of any commercial or financial relationships that could be construed as a potential conflict of interest.

## Footnotes

^1^http://www.csiro.au/files/files/p2ki.pdf

^2^http://www.plantstress.com/methods/index.asp

^3^See the “Plant Accelerator” at http://www.plantphenomics.org/TPA
